# A neural cell automated analysis system based on pathological specimens in a gerbil brain ischemia model

**DOI:** 10.1590/acb394224

**Published:** 2024-08-12

**Authors:** Eri Katsumata, Abhishek Kumar Ranjan, Yoshihiko Tashima, Takayuki Takahata, Toshiyuki Sato, Motoaki Kobayashi, Masami Ishii, Toyomi Takahashi, Asahi Oda, Momoko Hirano, Yoji Hakamata, Kazuhisa Sugai, Eiji Kobayashi

**Affiliations:** 1 Sysmex Co. – Hyogo, Japan.; 2 Sept.Sapie Co. – Tokyo, Japan.; 3 Nippon Veterinary and Life Science University – School of Veterinary Nursing and Technology – Department of Basic Science – Tokyo, Japan.; 4 Kobayashi Regenerative Research Institute – Wakayama, Japan.

**Keywords:** Deep Learning, Artificial Intelligence, Ischemia, Cerebral Cortex, Pathology

## Abstract

**Purpose:**

Amid rising health awareness, natural products which has milder effects than medical drugs are becoming popular. However, only few systems can quantitatively assess their impact on living organisms. Therefore, we developed a deep-learning system to automate the counting of cells in a gerbil model, aiming to assess a natural product’s effectiveness against ischemia.

**Methods:**

The image acquired from paraffin blocks containing gerbil brains was analyzed by a deep-learning model (fine-tuned Detectron2).

**Results:**

The counting system achieved a 79%-positive predictive value and 85%-sensitivity when visual judgment by an expert was used as ground truth.

**Conclusions:**

Our system evaluated hydrogen water’s potential against ischemia and found it potentially useful, which is consistent with expert assessment. Due to natural product’s milder effects, large data sets are needed for evaluation, making manual measurement labor-intensive. Hence, our system offers a promising new approach for evaluating natural products.

## Introduction

Histopathological diagnosis has traditionally relied on the expertise of pathologists specializing in the disease area. The morphological features observed under a microscope are often challenging to objectively describe or quantify. Accumulating a large number of cases or comparing them with other morphological data, as with routine clinical test data, has been extremely difficult. However, recent advancements in deep-learning technology, which has shown remarkable performance in image recognition, have prompted the development of numerical techniques for capturing the characteristics of pathological tissue images^1^^,^ 2.

In this study, we investigated whether pathology diagnosis could be automated using the sand rat unilateral cerebral ischemia model, which is highly effective as a cerebral ischemia induction model. Sand rats have congenital formation defects in the circle of Willis, making it easy and reproducible to induce cerebral ischemia by occluding the common carotid artery^3^. We have previously conducted research using this gerbil model to induce ischemic neuronal death in the ipsilateral cerebral hemisphere^4^^–^ 6. By comparing the number and morphology of neural cells in the cerebral cortex ischemic region with those that survived without ischemia, it is possible to observe changes within the same individual.

At first, we used properly processed specimens from gerbils with induced unilateral ischemia to count the remaining neural cells visually in the cerebral cortex ischemic region, creating a dataset. We then constructed a deep-learning model using this dataset and performed accuracy validation for the artificial intelligence (AI) counting system. Next, we used the completed AI system to compare the efforts and accuracy of the experienced pathologist and a pathology novice in microscopic assessments, demonstrating both the accuracy and time-saving benefits of the AI counting system. Finally, we compared and examined the accuracy of our developed AI-based pathology assessment method with past specimens^7^, in which manually counting all cells in the entire cerebral cortex region, which can number in the thousands, is extremely challenging. Especially in the brains of humans and large experimental animals, the labor required for manual visual counting is substantial. We have shown the potential for AI automation to replace this labor-intensive task of visual counting.

## Methods

### Sample preparation and imaging

**Figure 1:**
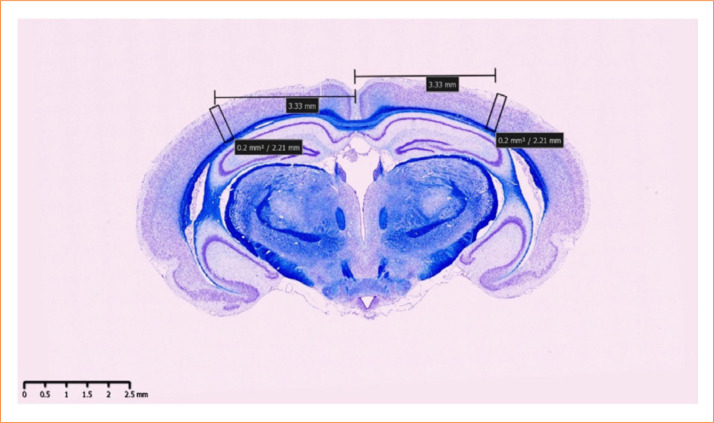
Specimen used in the study.

Source: Elaborated by the authors.

Paraffin blocks containing gerbil brains were repurposed from a prior study^7^. In brief, the samples were sectioned into 5-μm slices and stained with Klüver-Barrera. Each brain section was scanned using a NanoZoomer-SQ (Hamamatsu Photonics K.K., Japan) in × 40 mode (0.23 μm/pixel), and the images were saved using NDP.view software (NDPver2.0, Hamamatsu Photonics K.K., Japan) for quantifying the number of neurons in the cerebral cortex of each hemisphere. The measurement area in each hemisphere was defined as a 200-μm^2^ column between the interhemispheric and rhinal fissures (Fig. 1).

### Construction of the neural cell evaluation system with artificial intelligence capability

#### Data preparation and preprocessing

**Figure 2:**
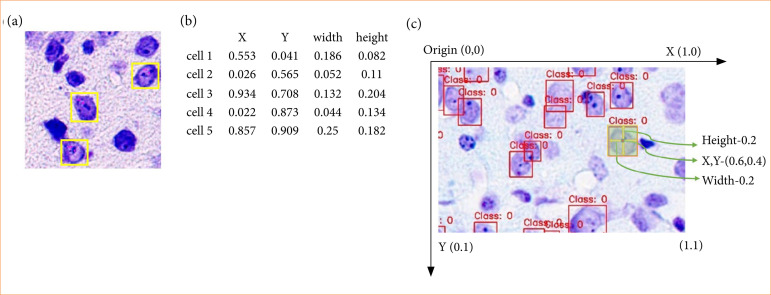
Model’s input information. **(a)** Sample annotations by an expert. **(b)** Sample annotation. **(c)** Annotation intuition.

Source: Elaborated by the authors.

**Figure 3:**
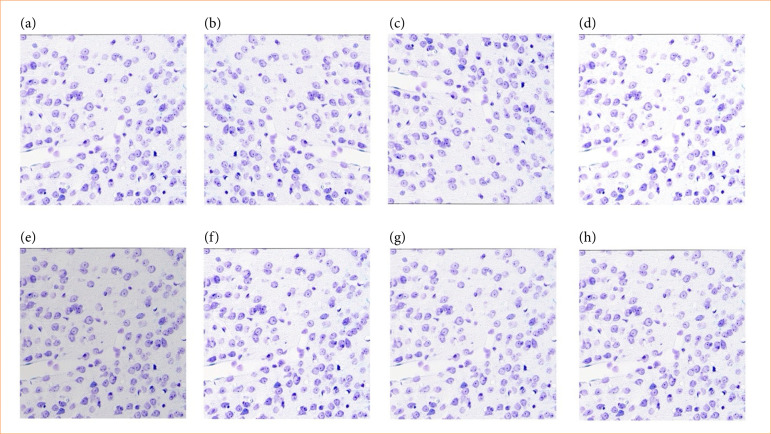
The output of data augmentation. **(a)** Original image. **(b)** Horizontal flip. **(c)** Vertical flip. **(d)** Positive brightness. **(e)** Negative brightness. **(f)** Positive Contrast. **(g)** Negative Contrast. **(h)** Sharpness.

Source: Elaborated by the authors.

Histopathology data of gerbil nerve cells, including sample annotations, were prepared. Expert-generated annotations contained images of cells with overlaid bounding boxes indicating the cells to be counted (Fig. 2a). These annotations were used to generate annotations for model training. Raw images contained two rectangular regions of interest in the left and right cortex regions (Fig. 1). To make them suitable for training, the left and right regions were cropped from the raw images at maximum resolution, resulting in exported images of approximately 8,000 × 2,000 pixels each. Annotations were prepared in the desired format for all images to create training data (Figs. 2b and 2c). Augmentation techniques, including horizontal and vertical flips, brightness adjustments of ± 20%, contrast variations of ± 10%, and sharpening by 5–10%, were applied to expand the training dataset (Fig. 3). Brightness adjustment modified overall lightness, contrast altered the contrast between light and dark areas, and sharpening enhanced image details.

#### Model selection

For our task, we opted for Detectron2^8^, an object detection library developed by Meta, among the available options. Detectron2 was chosen for its flexibility and cost-effectiveness. Its adaptability, customization options, extensive selection of pre-trained models, and ability to handle large images without post-processing make it a time-efficient and accurate choice for histopathology cell analysis. Its robustness in dealing with varying image resolutions ensures precise identification, making Detectron2 the optimal tool for the job.

#### Model training

Our primary objective is cell counting in the cortex region, for which cell localization suffices. Hence, we employed the Detectron2 detection algorithm to detect cells and subsequently count them. Among the pre-trained models offered by Detectron2 for object detection, we specifically selected the X101-FPN Faster R-CNN model^9^ due to its outstanding performance on the common objects in context (COCO) dataset^10^. This model employs the Faster R-CNN architecture, with the ResNext-101^11^ backbone playing a pivotal role in feature extraction. Additionally, the model incorporates the feature pyramid network^12^, which proves highly advantageous in effectively handling variations in image resolution during the object detection process.

#### Evaluation

We employed positive predictive value (PPV), sensitivity, and F1-score as evaluation metrics to assess the model’s performance. These metrics utilize the intersection over union (IoU) and confidence scores as thresholds.

Initially, a confidence score threshold was used to filter out low-probability detections. IoU, or the Jaccard index, measured the overlap between predicted bounding boxes and ground truth bounding boxes (annotated by experts). It was calculated as the ratio of the area of overlap between the two boxes to the area of their union. The IoU formula can be expressed as Eq. 1:


IoU=(Area of Overlap)/(Area of Union) (1)

Based on the IoU value, true positive detections had IoU values equal to or greater than a specified threshold. False positive detections were incorrect predictions with IoU values below the threshold, while false negatives referred to ground truth objects that were not detected. PPV represented the ratio of true positive detections to the total number of positive predictions, while sensitivity was the ratio of true positive detections to the total number of ground truth objects. F1-score was the harmonic mean of PPV and sensitivity.

**Figure 4:**
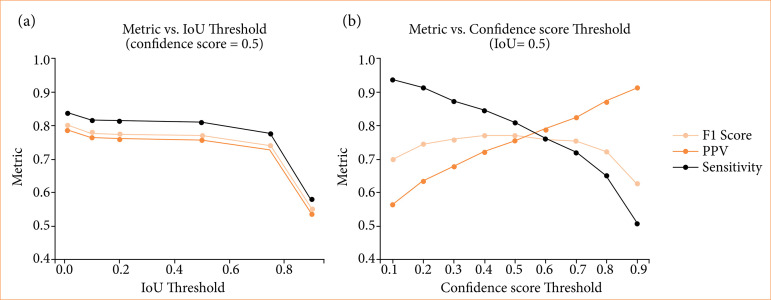
Threshold selection. Fix the value of one indicator and determine the appropriate threshold for the other indicator. **(a)** When confidence score is fixed. **(b)** When Intersection over Union (IoU) is fixed.

Source: Elaborated by the authors.

By adjusting the thresholds, we could control the trade off between PPV and sensitivity in our detection results. The suggested minimum IoU threshold for confirming a valid detection was 0.5, but selecting a higher value was more advantageous. Figure 4a illustrates the variation in metrics at various IoU thresholds, with a higher threshold resulting in decreased metric values. Therefore, 0.5 was chosen as the most optimal threshold.

Regarding the confidence score threshold (Fig. 4b), setting a higher threshold led to fewer detections, but with higher confidence in their accuracy, fewer false positives, and an increased PPV. Conversely, setting a lower threshold increased sensitivity, but it might include some false positives, potentially decreasing PPV. The maximum F1-score was achieved at a 0.5-confidence score threshold, and hence, it was selected as the threshold.

After the initial threshold selection, threshold values were optimized using additional trained models trained on different data splits. The final optimized threshold values remained at 0.5 for both confidence score and IoU, consistent with the initial threshold values. The average PPV, sensitivity, and F1-score from various models after threshold optimization were 0.77, 0.87, and 0.81 on the test dataset.

## Results

### Construction and performance evaluation of the artificial intelligence cell counting system

We assessed how well the results of the AI cell counting system (referred to as the AI system) matched the judgments made by skilled human observers. Among the thirty images analyzed, we used eighteen for training the AI system, adjusted its model parameters using four validation images to achieve optimal results, and evaluated its performance using eight test images. One of these test images exhibited staining artifacts, rendering it unsuitable for analysis, so it was excluded, leaving us with seven images for data analysis. Due to the variability in results based on the choice of images for training, validation, and testing, we conducted five trials with random combinations of these data splits and calculated the average values for each metric.

**Figure 5:**
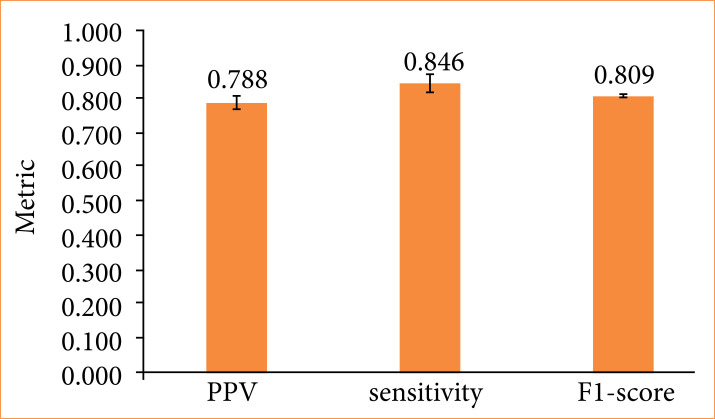
Positive predictive value, sensitivity, and F1-score in five different models. The results of the artificial intelligence system represent the averages of five trials, with error bars indicating one standard deviation.

Source: Elaborated by the authors.

In this evaluation, we considered regions identified as cells by visual inspection as ground truth. Regions identified as cells by the AI system that matched with the ground truth were counted as true positives, regions in which the AI system failed to detect cells were counted as false negatives, and regions where the AI system identified cells in areas not considered as cells in visual inspection were counted as false positives. Using true positives, false positives, and false negatives, we calculated precision (positive predictive value), sensitivity, and F1-score (Fig. 5).

When compared to the results of skilled pathologists, the AI system exhibited a precision of 79%, sensitivity of 85%, and an F1-score of 81%. Notably, precision appeared to be lower than sensitivity, suggesting that the AI system was more inclined to classify regions as cells that were not identified as such by the pathologists.

### Utility of cell counting in pathological images using the artificial intelligence system

#### Performance compared to novices

**Figure 6:**
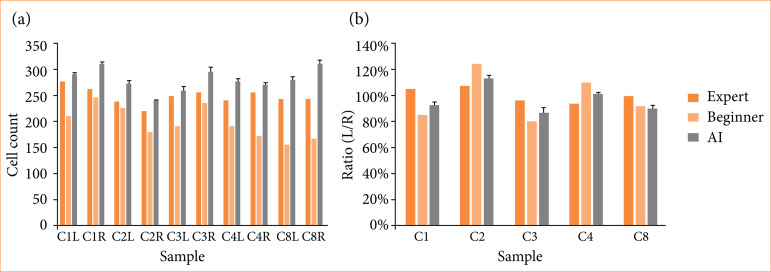
Comparison of cell counts by experts, novices, and the artificial intelligence (AI) system. Presented are the averages of five trials with error bars representing one standard deviation. **(a)** The cell counts. C1, C2, C3, C4, and C8 denote individual identifiers, while L and R indicate the left hemisphere and right hemisphere, respectively. **(b)** The left-right ratios obtained by dividing the cell counts in specific regions of the left hemisphere by those in the corresponding regions of the right hemisphere.

Source: Elaborated by the authors.

To demonstrate that the AI system we developed performs consistently, we compared its results with those of novice human observers. Figure 6a presents the cell counts by skilled pathologists, novice observers, and the AI system for ten images in the control group. Figure 6b shows the ratio of cell counts in the left hemisphere to those in the right hemisphere. The cell counts by novice observers were lower than those by skilled pathologists, whereas the AI system’s counts were generally higher than those of skilled pathologists. When examining the left-right ratio, for four out of five subjects, the AI system’s counts were closer to those of skilled pathologists than those of novice observers. Additionally, when we calculated the relative absolute error, novice observers exhibited an average error of 15%, whereas the AI system had an average error of 8%. These results suggest that the system can count cells more accurately than novices.

#### Time required for cell counting

**Figure 7:**
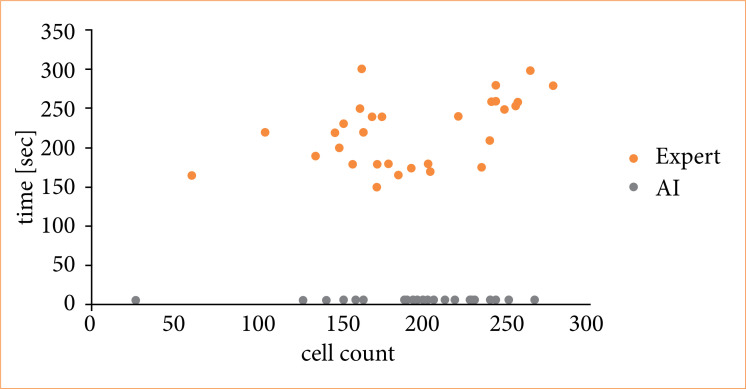
Relationship between the number of counted cells and the time taken for counting.

One of the advantages of using the AI system is the reduction in the time and effort required for cell counting. Figure 7 displays the number of cells counted and the time required for counting in the regions of interest. For counts by skilled pathologists, the time required increased as the number of cells to be counted increased. However, for counts by the AI system, the time required per image remained approximately 30 seconds, regardless of the number of cells. Moreover, in visual inspection, the same region might be counted multiple times, whereas the AI system, when using the same model (conditions), produced consistent results for the same region, allowing for evaluation in a single count.

AI: Artificial intelligence. Source: Elaborated by the authors.

#### Assessment of the effectiveness of treatment for brain ischemia using the artificial intelligence system

**Figure 8:**
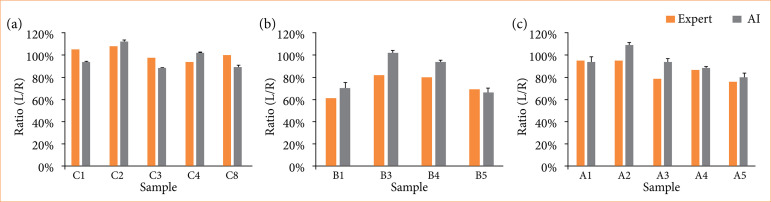
Left-right ratios in each group. The artificial intelligence (AI) results represent the averages of five trials, with error bars indicating one standard deviation. **(a)** Control group. **(b)** Group induced with ischemic neuronal cell death. **(c)** Group induced with ischemic neuronal cell death and administered hydrogen water.

Source: Elaborated by the authors.

We employed the AI system we developed to assess the effectiveness of interventions in histopathological samples from a sand rat model of cerebral ischemic neuronal death. We calculated the left-right ratios for the control group, the group with induced ischemic neuronal death, and the group with induced ischemic neuronal death treated with hydrogen water (Fig. 8).

Notably, one individual in the group with induced ischemic neuronal death exhibited staining artifacts and was excluded from the analysis. The average left-right ratios in each group were as follows: for the control group, skilled pathologists had a ratio of 101%, while the AI system had 98%; for the group with induced ischemic neuronal death, skilled pathologists had a ratio of 73%, while the AI system had 84%; and for the group with induced ischemic neuronal death treated with hydrogen water, skilled pathologists had a ratio of 86%, while the AI system had 94%. In each group, both skilled pathologists and the AI system exhibited differences in left-right ratios. Consequently, the system may be used to assess the effectiveness of interventions for brain ischemia.

## Discussion

In this study, we constructed an AI cell counting system to assess the effects of hydrogen water in a sand rat model and evaluated its potential applications.

The rapid fixation and transport of specimens have long been discussed in animal pathology. More recently, remote diagnosis of pathological specimens and the introduction of objective assessment methods using AI have been considered^13^.

**Figure 9:**
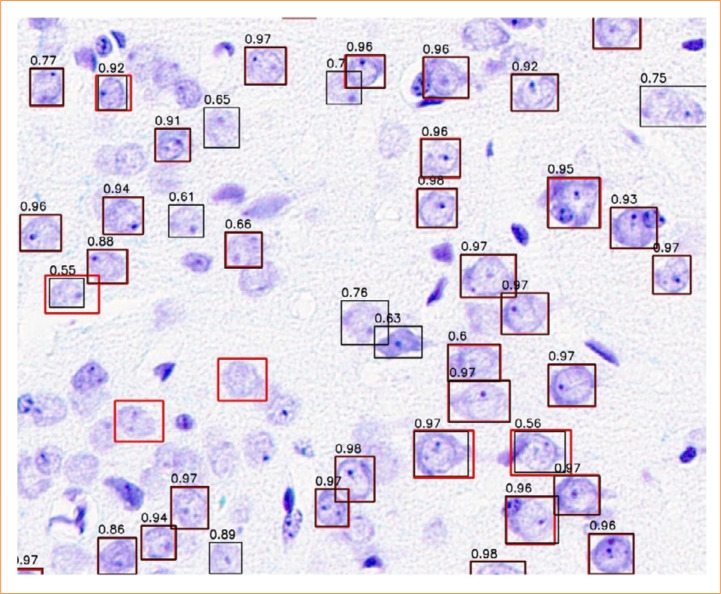
Regions detected as false positives. Red frames represent regions identified as cells by an expert, while black frames indicate regions identified as cells by the artificial intelligence system. The numerical values in black frames denote the confidence score for each region.

Source: Elaborated by the authors.

Considering these background factors, we examined the usefulness of a sand rat model of unilateral cerebral ischemia from the perspective of experimental animals and experimental pathology. First, we compared the performance of our constructed AI system with the results of skilled pathologists (Fig. 5). With a precision and sensitivity of 78% and 85%, respectively, it is suggested that there may be an excessive number of false positives at the current threshold. Figure 9 shows regions detected as false positives. Among these, there may be objects that require further examination by revisiting the visual inspection results of skilled pathologists. Although only one skilled pathologist conducted visual inspections in this study, involving multiple individuals in the examination process may improve the quality of the data generated by pathologists and further enhance the final model performance.

**Figure 10:**
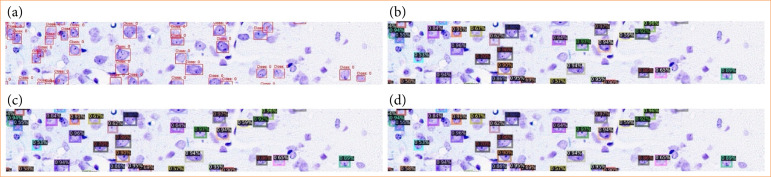
Variability in cell counts by the artificial intelligence system. Regions enclosed by squares were identified as cells. **(a)** Annotation results based on visual inspection. **(b-d)** Results of cell counts by the artificial intelligence system from the first to the third trial.

Source: Elaborated by the authors.

Next, we evaluated the utility of the AI cell counting system constructed in this study. The system’s features did not only include performance comparable to that of skilled pathologists and reduced counting time but also the potential to eliminate variability. When humans count the number of cells in a specific area, the results can vary depending on the observer and can even vary when the same observer counts the same area multiple times^14^. In contrast, the AI system produces consistent results for the same region when using the same model (Fig. 10). Therefore, by constructing a high-performance model that reflects the results of counts by skilled pathologists, it is likely that highly accurate and consistent cell counting results can be obtained.

Lastly, we assessed whether the AI system we constructed could be used as an application to determine the effectiveness of interventions. We used pathological specimens from a sand rat model of unilateral cerebral ischemia that had already been reported^7^. Our AI system showed results similar to the microscopic results of skilled pathologists in this study.

## Conclusion

Generally, non-pharmaceuticals like hydrogen water do not have a significant impact on experimental pathology results compared to pharmaceuticals^15^. In previously reported pathological studies of sand rat models, the differences observed between control groups were minimal, and, to detect these subtle differences, further investigation with a larger sample size was required^7^. Furthermore, evaluating pathological specimens in the same area multiple times from large animals extrapolated to humans requires a significant amount of effort^15^. Therefore, the approach using the AI system constructed in this study allows for the analysis of a large amount of data with minimal human effort, and it is expected that accurate and objective evaluation systems can be developed in the field of functional foods and health foods^16^.

## Data Availability

The data will be available upon reasonable request.
